# The Prognostic Significance of Circulating Tumor Cells in Patients with Pancreatobiliary Cancer

**DOI:** 10.5152/tjg.2023.22260

**Published:** 2023-03-01

**Authors:** İlkay Tuğba Ünek, İlhan Öztop, Yasemin Başbınar, Tarkan Ünek, Asım Leblebici, Caner Karaca, Ece Çakıroğlu, Tuğba Uysal, Anıl Aysal, Hülya Ellidokuz, Özgül Sağol, İbrahim Astarcıoğlu

**Affiliations:** 1 Department of Medical Oncology, Dokuz Eylül University Faculty of Medicine, İzmir, Turkey; 2 Department of Translational Oncology, Dokuz Eylül University, Institute of Oncology, İzmir, Turkey; 3 Department of General Surgery, Dokuz Eylül University, School of Medicine, Izmir, Turkey; 4 Department of Translational Oncology, Dokuz Eylül University, Institute of Health Sciences, İzmir, Turkey; 5 Department of Genomics and Molecular Biotechnology, Dokuz Eylül University, İzmir International Biomedicine and Genome Institute, İzmir, Turkey; 6 Department of Molecular Biology and Genetics, Hitit University Faculty of Science and Art, Çorum, Turkey; 7 Department of Pathology, Dokuz Eylül University Faculty of Medicine, İzmir, Turkey; 8 Department of Preventive Oncology, Dokuz Eylül University, Institute of Oncology, İzmir, Turkey

**Keywords:** Circulating tumor cells, pancreatobiliary cancer, prognosis

## Abstract

**Background:**

Circulating tumor cells are cancer cells which separate from the primary tumor and enter systemic circulation. In this study, it was aimed to examine the relationship between circulating tumor cells isolated and identified from the peripheral blood of patients with pancreatobiliary cancer, with the clinicopathological characteristics of the patients and their overall survival.

**Methods:**

A total of 21 patients were included in the study. Density-gradient centrifugation with the OncoQuick® assay was performed for isolation of circulating tumor cells from peripheral blood. In order to identify circulating tumor cells, enriched samples underwent flow cytometric analysis.

**Results:**

The rate of patients with positive surgical margin in the high circulating tumor cell group (circulating tumor cell >15) was identified to be statistically significantly high compared to the group with low circulating tumor cells (circulating tumor cell ≤15) (83.3% vs. 16.7%; *P* = .041). Median neutrophil/lymphocyte ratio was found to be higher in the high circulating tumor cell group compared to the low circulating tumor cell group, which was close to statistical significance (2.37 vs. 1.41; *P *= 0.055).

**Conclusions:**

Circulating tumor cells were identified to have a significant relationship with surgical margin positivity in our study for the first time, suggesting that the circulating tumor cells count in peripheral blood in preoperative patients may be a biomarker predicting positive surgical margin. Due to the very low number of studies assessing the relationship between circulating tumor cells and neutrophil/lymphocyte ratio, our study which identified relationship close to statistical significance between circulating tumor cells and neutrophil/lymphocyte ratio, significantly contributes to the literature on the topic of the possible role of lymphocytes in circulating tumor cell clearance.

**Main Points**

- Considering circulating tumor cell (CTCs) as potential sources of systemic recurrence in cancer patients, isolation and identification of these cells in cancers with high postoperative systemic recurrence risk like pancreatobiliary cancer has great importance.
- In the literature, CTCs were identified to have a significant relationship with surgical margin positivity in our study for the first time, suggesting that the CTC count in peripheral blood in preoperative patients may be a biomarker predicting positive surgical margin.
- Due to the very low number of studies assessing the relationship between CTCs and neutrophil/lymphocyte ratio (NLR), our study which identified relationship close to statistical significance between CTCs and NLR, significantly contributes to the literature on the topic of the possible role of lymphocytes in CTC clearance.

## Introduction

In spite of developments in recent years in surgical techniques and perioperative treatment methods, the prognosis of pancreatobiliary cancers (PBCs) is still very poor. In terms of rankings in cancer-related deaths, pancreatic cancer is in fourth place, while biliary tract cancers are in fifth place in men and seventh place in women.^1^ Most PBC patients are in local advanced or metastatic stage at the time of diagnosis. Operated patients develop high rates of recurrence in the early postoperative period. The tumors are resistant to chemotherapy and radiotherapy. As a result, there is a need for reliable biomarkers to ensure early diagnosis and early detection of tumor recurrence and to determine prognosis and most effective treatment.^2,3^

Circulating tumor cells (CTCs) are cancer cells which separate from the primary tumor and enter systemic circulation. Circulating tumor cells are thought to be responsible for formation of metastasis in distant organs. Circulating tumor cells isolated from simple peripheral blood samples may ensure early detection of cancer and provide information about current biological activity of cancer, newly emerging mutations, and chemotherapy resistance. Circulating tumor cells may be used as prognostic biomarkers and treatment targeting CTCs may reduce systemic recurrence and lengthen patient survival.^4^ However, there is 1 CTC for every 10^5^ blood cells in peripheral blood. Isolation of CTCs from blood is technically difficult due to the very low number of tumor cells compared to the excess number of blood cells in circulation. There is a need for enrichment and detection methods to count CTCs. Among methods providing isolation of these rare cells from peripheral blood, the immunomagnetic method using antibodies to bind the surface antigens, density-gradient-centrifugation, and size-based isolation methods which are methods using physical properties can be listed. After CTC isolation, CTC identification is performed. Among current identification methods are immunohistochemical analyses using monoclonal antibodies, nucleic acid-based methods like polymerase chain reaction, and cytometric methods like flow cytometry.^6,7^ The CellSearch system ensuring immunomagnetic isolation and immunostaining identification of CTCs in peripheral blood is the only platform approved by the U.S. Food and Drug Administration to be used in clinical practice to estimate prognosis in metastatic breast, colon, and prostate cancer patients.^6,7^

There is no standard CTC isolation/identification method for pancreatic and biliary cancer patients and there is contradictory information about the prognostic role of CTCs in the literature.^7-14^ As a result, there is a need for development of methods for isolation and identification of CTCs in PBC and prospective studies about the place of CTCs in clinical practice.^7-12^ In this study, it was aimed to examine the relationship between CTCs isolated and identified from the peripheral blood of patients with PBC, with the clinicopathological characteristics of the patients and their overall survival (OS).

## Materials and Methods

### Patients

A total of 21 patients who were scheduled for operation or systemic treatment with the diagnosis of biliary tract, pancreatic, and ampullary carcinoma in the Departments of General Surgery and Medical Oncology of Dokuz Eylül University Faculty of Medicine were included in the study. We obtained patients informed consent and approval of the ethical committee of Dokuz Eylül University for this study (date: June 26, 2014; decision no: 2014/23-21).

### Blood Samples

Patients with operation planned had peripheral blood samples taken before operation, while metastatic patients with systemic chemotherapy planned had peripheral blood samples taken before chemotherapy. For CTC analysis, 8 cc peripheral blood was placed in anticoagulant tubes and processed within 2 hours after collection.

### Circulating Tumor Cell Enrichment and Identification

Density-gradient centrifugation with the OncoQuick® assay was performed according to the standard protocol provided by the manufacturer (Grenier BioOne, Frickenhausen, Germany). Peripheral blood samples of 8 mL, previously cooled on ice, were poured into precooled OncoQuick® tubes and centrifuged for 20 minutes at 1600 g at 4°C. After centrifuging, the entire volume of interface cells in the compartment between the uppermost plasma and lowermost separation medium were transferred to a new centrifuge tube. These cells were centrifuged with washing buffer at 200 g for 10 minutes at 4°C. Without damaging the cell pellet formed after centrifugation, as much supernatant as possible was aspirated. The cell pellet was resuspended in phosphate-buffered saline and stored at –145°C until flow cytometric analysis.

In order to identify CTCs, enriched samples underwent flow cytometric analysis on Navios EX Flow Cytometer (O’Callaghan’s Mill, Beckman Coulter, Ireland) using Kaluza Analysis 2.1 software (O’Callaghan’s Mill, Beckman Coulter, Ireland). The samples were stained with CD326 [epithelial cell adhesion molecule (EpCAM)] monoclonal antibody (clone 1B7) conjugated with phycoerythrin (PE) and CD45 monoclonal antibody (clone HI30) conjugated with PE-Cyanine7. Mouse IgG1 kappa isotype control (clone P3.6.2.8.1), PE and mouse IgG1 kappa isotype control (clone P3.6.2.8.1), and PE-Cyanine7 were used as isotype controls to minimize the influence of nonspecific bindings on gating and background. All antibodies were purchased from eBioscience^TM^. The cells were sorted according to their CD45 and EpCAM characters. The cells characterized by CD45 negative and EpCAM-positive signals were defined as CTCs.

### Statistical Analysis

Analysis of data was completed with the Statistical Package for Social Sciences version 24.0 software (IBM Corp.; Armonk, NY, USA). Comparison of 2 independent groups used the nonparametric Mann–Whitney *U*-test, comparison of more than 2 groups used the Kruskal–Wallis test, while comparison of categoric variables used the Fisher’s exact test. Kaplan–Meier log-rank (Mantel–Cox) analysis was used to investigate the effect of factors on mortality and survival. Parameters significant on univariate analysis were assessed with multivariate Cox regression analysis. Quantitative data are shown in tables as mean ± SD and median (minimum–maximum), with categoric data given as number (n) and percentage (%). Data were investigated in the 95% CI with *P*-values smaller than .05 accepted as significant.

## Results

Our study included 21 patients with PBC. The clinicopathological characteristics of the patients are shown in Table 1. Median age was 61.0 years (range: 40.0-83.0). Of patients, 66.7% were male and 33.3% were female. Among patients, 57.1% had pancreatic carcinoma, 19.0% had biliary tract carcinoma, and 23.8% had ampullary carcinoma. Thirteen patients (61.9%) had pancreaticoduodenectomy (Whipple) operation, 3 patients (14.3%) had distal pancreatectomy operation, and 3 patients (14.3%) had segmental liver resection. Two patients (9.5%) were not operated due to metastatic disease. The operated patients had median tumor diameter of 2.8 cm (range: 1.0-7.0), with 57.1% of patients having tumor size ≤3 cm. While 23.8% of patients did not have lymph node (LN) metastasis, 38.1% had 1-3 LN metastasis and 28.6% had ≥4 LN metastasis. A total of 71.4% of patients’ tumors were well-differentiated and 61.9% of patients had negative surgical margin. Peripheral blood samples taken before operation in operated patients and before chemotherapy in metastatic patients had median values identified as carbohydrate antigen 19-9 (Ca 19-9) 23.2 U/mL (range: 0.8-4880.0), CTCs 15 (range: 1-61), leukocyte 6900/μL (range: 5100-11300), neutrophil 4200/μL (range: 800-6500), lymphocyte 2100/μL (range: 1200-4200), and monocyte 600/μL (range: 400-1500).

According to clinicopathological characteristics of patients, median CTC counts are shown in Table 2. There was no difference in median CTC counts according to patient age, sex, tumor type, tumor size, LN metastasis numbers, histologic differentiation, perineural invasion, and lymphovascular invasion. The median CTC counts were identified to be statistically significantly high in patients with positive surgical margin compared to those with negative surgical margin (30 vs. 9; *P *= .043). When patients were grouped according to leukocyte, neutrophil, lymphocyte, and monocyte counts and neutrophil/lymphocyte ratio (NLR) median values, those with NLR >2 had higher median CTC counts compared to those with NLR ≤2, however, this did not reach statistical significance (19 vs. 7; *P *= .090).

Patients were divided into 2 groups according to CTC counts as the low CTC group (CTCs ≤15) and high CTC group (CTCs >15) and clinical and pathological features were compared (Table 3). Among patients with negative surgical margin, 76.9% were in the low CTC group and 23.1% were in the high CTC group. For patients with positive surgical margin, 16.7% were in the low CTC group and 83.3% were in the high CTC group (*P *= .041). Median NLR was identified to be high at almost statistically significant level in the high CTC group compared to the low CTC group (2.37 vs. 1.41; *P *= .055). However, perhaps due to small sample size, there was no significant relationship identified between other clinicopathological variables and CTC count (Tables 2 and 3).

Mean follow-up was 17.5 months (range: 0.2-42.2) and 15 of the 21 (71.4%) patients died. Patients had median OS of 12.7 months (0.0-27.3). Univariate Cox regression analysis was performed with the aim of researching factors determining OS (Table 4). Compared to patients aged ≤65 years, patients >65 years were identified to have shorter median OS (23.2 months vs. 4.2 months; *P *= .003). Compared to patients with good histological differentiation, patients with moderate/poor histologic differentiation were identified to have shorter median OS (21.6 months vs. 4.2 months; *P *= .043).

In terms of median OS, though not at statistical significance, it was shorter for those with LN involvement >3 compared to those with ≤3 involvement (5.6 months vs. 18.3 months; *P *= .312); in those with positive surgical margin compared to those with negative surgical margin (7.5 months vs. 21.6 months; *P *= 0.319); in those with Ca 19-9 >35 U/mL compared to those with Ca 19-9 ≤35 U/mL (7.5 months vs. 21.6 months; *P *= .368); and in those with median CTC >15 compared to those with CTC ≤15 (8.5 months vs. 18.3 months; *P *= 0.779) (Table 4). This is thought to be due to the low number of patients in our study. In multivariate analysis, independent predictors of OS were age and histologic differentiation (Table 5).

## Discussion

In our study, patients with positive surgical margin were identified to have statistically significantly higher median CTC counts compared to those with negative surgical margin (30 vs. 9; *P *= .043) (Table 2). Similarly, the rate of patients with positive surgical margin in the high CTC group (CTC >15) was identified to be statistically significantly high compared to the group with low CTC (CTC ≤15) (83.3% vs. 16.7%; *P* = 0.041) (Table 3).

There are several studies assessing the relationship between CTCs and surgical margin positivity of patients with PBC.^15-20^ To the best of our knowledge, the first study to identify a significant relationship between surgical margin status and CTC counts in PBC patients is our study. Soeth et al^15^ showed that CTC positivity rate was associated with LN metastasis, distant metastasis, and short survival in patients operated for pancreatic cancer. They did not identify a significant difference in CTC positivity rate according to surgical margin status. Vicente et al^16^ identified similar surgical margin positivity rates in operated pancreatic cancer patients with low numbers of CTC identified and patients with high numbers of CTC identified. Similarly, other studies including pancreatic cancer patients did not show a significant correlation between CTC and surgical margin status.^17-20^ In PBCs, Ca 19-9 level, advanced tumor stage, LN metastasis, tumor localization, and tumor size are clinical parameters associated with surgical margin positivity.^21-23^ The significant relationship between CTC counts and surgical margin positivity identified in our study leads to the consideration that the CTC count in peripheral blood in preoperative patients may be a biomarker predicting surgical margin positivity. High preoperative CTC counts may be a warning sign for surgeons and pathologists evaluating intra-operative frozen section in terms of surgical margin positivity.

In our study, there was a relationship close to statistical significance between CTCs and NLR. Median NLR was identified to be higher and almost at statistical significance in the group with high CTC compared to the group with low CTC (2.37 vs. 1.41; *P* = .055) (Table 3). Compared with patients with CTC ≤15, patients with CTC >15 were identified to have lower lymphocyte counts, however, this did not reach statistical significance (2400/μL vs. 1800/μL; *P* = .514) (Table 3). Similar to our study, a study investigating the clinicopathological characteristics of pancreatic cancer patients according to CTC status found that patients with CTC (+) had significantly lower lymphocyte counts and significantly higher NLR compared to those who were CTC (-). Leukocyte, neutrophil, and monocyte counts were not different between the CTC groups. The results of this study showed that lymphocytes may play an important role in clearance of CTCs.^24^ However, Chang et al^13^ identified no correlation between CTC counts with lymphocyte counts and NLR in pancreatic cancer patients. In the literature, there are very few studies assessing the correlations between CTCs with leukocyte, neutrophil, lymphocyte and monocyte counts, and NLR.^13,24,25^ In our study, the high CTC group had NLR results that were high and close to statistical significance which supports the idea that lymphocytes may play an important role in CTC clearance. There is a need for more studies on this topic.

In the literature, there are contradictory results from studies assessing the role of CTCs in determining survival of PBC patients. The reason for this is that these studies used different methods, on different platforms, obtaining CTC subtypes with different biological features. Studies using the same CTC detection platforms may even obtain incompatible results.^14^ A study using the density-gradient centrifugation method identified a correlation between CTC and survival;^15^ however, 2 other studies using the same method did not identify a correlation between CTC and survival.^26,27^ In our study using the density-gradient centrifugation method, patients with CTC >15 were identified to have shorter median OS compared to those with CTC ≤15 (8.5 months vs. 18.3 months); however, this did not reach statistical significance (Table 4). In studies using the size-based isolation method, there are those identifying a CTC and survival correlation,^17,18,24,28,29^ just as there are studies published which did not identify a correlation with survival.^10,19,30,31^ Among studies using the immunomagnetic method, there are studies showing a correlation of CTC with survival,^8,9,20,25,32-36^ just as there are studies showing no correlation with survival.^11,37^ Two studies using the anti-EpCAM-coated microfluidic system identified a correlation of CTC with survival,^38,39^ while another study using the same method did not identify a correlation of CTC with survival.^13^ Two studies detecting CTC with the EpCAM-independent subtraction enrichment and immunostaining-fluorescence in situ hybridization method showed a significant correlation between CTC positivity with survival^40,41^; however, another study using the same method did not identify a relationship between CTC and survival.^14^

The only study using the same CTC isolation method (OncoQuick® density-gradient centrifugation) and same CTC identification method (flow cytometry) with our study was published by Sergeant et al.^42^ They isolated and identified CTCs in peripheral blood from 10 pancreatic cancer patients and investigated gene expressions with microarray analysis. They found overexpression of transforming growth factor β1, cytosolic phospholipase A2, MYC-associated factor X, and 9 other genes related to both p38 mitogen-activated protein kinase signaling and cell motility in circulating cancer cells. They identified that overexpression of these newly defined cell motility gene signature in primary tumor of 78 pancreatic cancer patients was an independent determinant of survival.^42^

The epithelial-mesenchymal transition (EMT) involves many molecular and cellular changes with reduction of epithelial proteins, negativity of EpCAM expression, and increased mesenchymal proteins. Cancer cells with EMT phenotype are especially associated with cancer metastasis, progression, and treatment resistance and are accepted as very malignant.^24^ In our study with CTC identification based on the EpCAM-based CTC detection method, the inability to identify EpCAM negative cancer cells may be a reason for our inability to show a correlation between CTC and survival. Cancer stem cells (CSC) have the ability to initiate tumor growth and renew and sustain themselves due to stem cell-like properties and are thought to be responsible for distant metastasis, tumor recurrence, chemotherapy, and radiotherapy resistance. Circulating CSCs comprise a small proportion of CTCs and carry both EMT and CSC phenotypes.^43^ Studies have identified that elevations in the level of CSC in circulation in cancer patients is associated with poor prognosis.^17,44^ It is considered that the CTC phenotype, rather than the number of CTC, may play a more important role in determining prognosis in patients.^17^

Limitations of our study include the low number of patients and the lack of determination of CTC phenotypes. There is a need for studies including more patients, determining CTC phenotypes in addition to CTC counts, and comparing prognosis between these phenotype subgroups. Determination of CTC phenotype associated with poor prognosis will allow the opportunity to develop targeted treatment for CTCs with this phenotype and lengthen survival for patients.

In conclusion, considering CTCs as potential sources of systemic recurrence in cancer patients, isolation and identification of these cells in cancers with high postoperative systemic recurrence risk like PBC has great importance. In the literature, CTCs were identified to have a significant relationship with surgical margin positivity in our study for the first time, suggesting that the CTC count in peripheral blood in preoperative patients may be a biomarker predicting positive surgical margin. Due to the very low number of studies assessing the relationship between CTCs and NLR, our study which identified relationship close to statistical significance between of CTCs and NLR, significantly contributes to the literature on the topic of the possible role of lymphocytes in CTC clearance.

## Figures and Tables

**Table 1.** Clinicopathological Characteristics of Patients

Characteristics	n	%
Age, median (range)	61.0 (40.0-83.0)	
Gender		
Male	14	66.7
Female	7	33.3
Tumor type		
Pancreatic cancer	12	57.1
Biliary tract cancer	4	19.0
Ampullary cancer	5	23.8
Surgery		
Pancreaticoduodenectomy (Whipple)	13	61.9
Distal pancreatectomy	3	14.3
Segmental liver resection	3	14.3
No surgery	2	9.5
Stage^†^		
T2, N0, M0	2	9.5
T3, N0, M0	3	14.3
T1-3, N1, M0	12	57.1
T1-3, N2/T4, AnyN, M0	2	9.5
AnyT, AnyN, M1	2	9.5
Maximum tumor diameter, median (range)	2.8 (1.0-7.0)	
Tumor in greatest dimension		
Tumor ≤ 3 cm	12	57.1
Tumor > 3 cm	7	33.3
NA	2	9.5
LN involvement		
LN negative	5	23.8
1-3 LN positive	8	38.1
≥4 LN positive	6	28.6
NA	2	9.5
Histologic differentiation		
Well	15	71.4
Moderate	2	9.5
Poor	4	19.0
Surgical margin		
Negative (R0)	13	61.9
Positive (R1)	6	28.6
NA	2	9.5
Perineural invasion	13	61.9
Lymphovascular invasion	15	71.4
Ca 19-9,^‡^ median (range)	23.2 (0.8-4880.0)	
CTCs,* median (range)	15 (1-61)	

^†^Stages according to the American Joint Committee on Cancer (AJCC) Tumor Node Metastasis (TNM) Staging (8th ed., 2017).

^‡^U/mL.

^*^8 mL of venous blood.

Ca 19-9, carbohydrate antigen 19-9; CTCs, circulating tumor cells; LN, lymph node; NA, not applicable.

**Table 2.** The Median Number of CTCs According to Clinicopathological Characteristics of Patients

Characteristics	CTCs/8 mL, median (Range)	*P*
Age		
≤65	11 (1-61)	.116
>65	32 (2-42)	
Gender		
Male	15 (1-61)	.432
Female	8 (2-32)	
Tumor type		
Pancreatic cancer	15 (1-42)	.838
Biliary tract cancer	13 (5-61)	
Ampullary cancer	13 (2-19)	
Tumor in greatest dimension		
Tumor ≤3 cm	11 (1-42)	.351
Tumor >3 cm	21 (1-61)	
LN involvement		
≤3 LN positive	15 (1-61)	0.93
>3 LN positive	14 (2-35)	
Histologic differentiation		
Well	15 (1-61)	.969
Moderate/Poor	12 (2-35)	
Surgical margin		
Negative (R0)	9 (1-61)	**.043**
Positive (R1)	30 (5-42)	
Perineural invasion		
Negative	11 (2-21)	.312
Positive	19 (1-61)	
Lymphovascular invasion		
Negative	17 (2-42)	.688
Positive	15 (1-61)	
Ca 19-9		
≤35 U/mL	11 (1-61)	.346
>35 U/mL	15 (2-42)	
Leukocyte		
≤6900/μL	9 (1-61)	.269
>6900/μL	15 (2-42)	
Neutrophil		
≤4200/μL	12 (1-61)	.765
>4200/μL	15 (1-42)	
Lymphocyte		
≤2100/μL	15 (1-61)	1
>2100/μL	14 (1-42)	
Monocyte		
≤600/μL	13 (1-61)	.558
>600/μL	15 (5-42)	
Neutrophil/lymphocyte ratio		
≤2	7 (1-38)	.09
>2	19 (1-61)	
C-reactive protein		
≤5 mg/L	13 (1-38)	.521
>5 mg/L	15 (1-61)	

Ca 19-9, carbohydrate antigen 19-9; CTCs, circulating tumor cells; LN, lymph node.

**Table 3.** Relationship Between CTC Status and Clinicopathological Characteristics

Characteristics	CTCs/8 mL ≤15 Median or %	Range	CTCs/8 mL >15 Median or %	Range	*P*
Age, median	58	40-83	64	53-79	.275
Gender					
Male	57.1		42.9		.656
Female	71.4		28.6		
Tumor type					
Pancreatic cancer	58.3		41.7		.607
Biliary tract cancer	50		50		
Ampullary cancer	80		20		
Tumor diameter, median	2	1.0-5.0	3	1.5-7.0	.481
Tumor diameter					
Tumor ≤ 3 cm	66.7		33.3		.377
Tumor > 3 cm	42.9		57.1		
LN involvement					
LN negative	40		60		.603
LN positive	64.3		35.7		
LN involvement					
≤3 LN positive	61.5		38.5		1
>3 LN positive	50		50		
Histologic differentiation					
Well	60		40		1
Moderate/poor	66.7		33.3		
Surgical margin					
Negative (R0)	76.9		23.1		**.041**
Positive (R1)	16.7		83.3		
Perineural invasion					
Negative	83.3		16.7		.177
Positive	46.2		53.8		
Lymphovascular invasion					
Negative	50		50		1
Positive	60		40		
Ca 19-9, median (U/mL)	20.8	5.7-262.9	41.3	0.8-4880	.447
Leukocyte (/μL)	6600	5100-11 300	7100	6300-10 000	.261
Neutrophil (/μL)	4100	800-6500	4200	3700-6500	.167
Lymphocyte (/μL)	2400	1200-4200	1800	1500-2900	.514
Monocyte (/μL)	500	400-1500	600	400-900	.578
NLR	1.41	0.19-3.75	2.37	1.45-3.82	**.055**
C-reactive protein (mg/L)	6.2	0.6-31.2	5.55	0.5-21.2	.515

Ca 19-9, carbohydrate antigen 19-9; CTCs, circulating tumor cells; LN, lymph node; NLR, neutrophil/lymphocyte ratio.

**Table 4.** Univariate Analysis for Overall Survival

Characteristics	Median OS (Months)	95% CI	Log-Rank *P*
Age			
≤65	23.2	8.8-37.7	**.003**
>65	4.2	0.0-13.8	
Gender			
Male	12.7	0.0-30.6	.74
Female	12.1	0.0-28.7	
Tumor type			
Pancreatic cancer	8.5	0.0-17.3	.842
Biliary tract cancer	23.2	3.1-43.4	
Ampullary cancer	12.1	0.0-35.5	
Tumor diameter			
Tumor ≤3 cm	12.1	0.0-30.5	.993
Tumor >3 cm	12.7	2.0-23.4	
LN involvement			
≤3 LN positive	18.3	5.2-31.4	.312
>3 LN positive	5.6	0.5-10.8	
Histologic differentiation			
Well	21.6	8.3-35.0	**.043**
Moderate/poor	4.2	0.0-13.0	
Surgical margin			
Negative (R0)	21.6	8.6-34.7	.319
Positive (R1)	7.5	4.0-10.9	
Ca 19-9			
≤35 U/mL	21.6	14.0-29.3	.368
>35 U/mL	7.5	2.1-12.8	
CTCs/8 mL			
CTCs ≤15	18.3	1.5-35.1	.779
CTCs >15	8.5	1.3-15.7	

Ca 19-9, carbohydrate antigen 19-9; CTCs, circulating tumor cells; LN, lymph node.

**Table 5.** Multivariate Analysis for Overall Survival

Parameters	Coefficient *β*	Standard Error	*P*	Odds Ratio	95% CI
Age	1.945	0.625	.002	6.991	2.052-23.817
Histologic differentiation	1.714	0.635	.007	5.549	1.598-19.270
